# Decreases in Molecular Diffusion, Perfusion Fraction and Perfusion-Related Diffusion in Fibrotic Livers: A Prospective Clinical Intravoxel Incoherent Motion MR Imaging Study

**DOI:** 10.1371/journal.pone.0113846

**Published:** 2014-12-01

**Authors:** Pu-Xuan Lu, Hua Huang, Jing Yuan, Feng Zhao, Zhi-Yi Chen, Qinwei Zhang, Anil T. Ahuja, Bo-Ping Zhou, Yì-Xiáng J. Wáng

**Affiliations:** 1 Department of Radiology, The Shenzhen No. 3 People's Hospital, Guangdong Medical College, Shenzhen, China; 2 Department of Imaging and Interventional Radiology, Prince of Wales Hospital, The Chinese University of Hong Kong, Shatin, New Territories, Hong Kong SAR; 3 Laboratory of Ultrasound Molecular Imaging, The Third Affiliated Hospital of Guangzhou Medical University, Guangzhou, China; 4 Department of Hepatology, The Shenzhen No. 3 People's Hospital, Guangdong Medical College, Shenzhen, China; Affiliated Hospital of North Sichuan Medical College, China

## Abstract

**Purpose:**

This study was aimed to determine whether pure molecular-based diffusion coefficient (*D*) and perfusion-related diffusion parameters (perfusion fraction *f*, perfusion-related diffusion coefficient D*) differ in healthy livers and fibrotic livers through intra-voxel incoherent motion (IVIM) MR imaging.

**Material and Methods:**

17 healthy volunteers and 34 patients with histopathologically confirmed liver fibrosis patients (stage 1 = 14, stage 2 = 8, stage 3& 4 = 12, METAVIR grading) were included. Liver MR imaging was performed at 1.5-T. IVIM diffusion weighted imaging sequence was based on standard single-shot DW spin echo-planar imaging, with ten *b* values of 10, 20, 40, 60, 80, 100, 150, 200, 400, 800 sec/mm^2^ respectively. Pixel-wise realization and regions-of-interest based quantification of IVIM parameters were performed.

**Results:**

*D, f*, and D* in healthy volunteer livers and patient livers were 1.096±0.155 vs 0.917±0.152 (10^−3^ mm^2^/s, p = 0.0015), 0.164±0.021 vs 0.123±0.029 (p<0.0001), and 13.085±2.943 vs 9.423±1.737 (10^−3^ mm^2^/s, p<0.0001) respectively, all significantly lower in fibrotic livers. As the fibrosis severity progressed, *D*, *f*, and D* values decreased, with a trend significant for *f* and D*.

**Conclusion:**

Fibrotic liver is associated with lower pure molecular diffusion, lower perfusion volume fraction, and lower perfusion-related diffusion. The decrease of *f* and D* in the liver is significantly associated liver fibrosis severity.

## Introduction

Chronic liver disease is a major public health problem worldwide [1]. Liver fibrosis, a common feature of almost all chronic liver diseases, involves the accumulation of collagen, proteoglycans, and other macromolecules in the extracellular matrix. Clinically liver fibrosis usually has an insidious onset and progresses slowly over decades. Originally considered to be irreversible, hepatic fibrosis is now regarded as a dynamic process with the potential for regression [1]. To date, noninvasive diagnostic tests available from clinical practice are not sensitive or specific enough to detect occult liver injury at early or intermediate stages. The reliability of liver function tests, serological tests of specific serum makers, and liver stiffness measurement in assessing liver fibrosis is still under investigation [2]–[6]. Liver biopsy is currently the standard of reference for the diagnosis and staging of liver fibrosis. However, it is an invasive procedure with possible complications [7]. Histologic assessment of fibrosis is also an inherently subjective process, and it is subject to sampling variability. The extent of variations from observer interpretation by expert histopathologists may be as high as 20% [8]. These limitations make liver biopsy somewhat suboptimal for diagnosis and longitudinal monitoring in the general population. A noninvasive and quantitative technique for assessing liver fibrosis and monitoring disease progression or therapeutic intervention will be valuable [1], [9], [10].

The principle of intra-voxel incoherent motion (IVIM) imaging is a method initially proposed by Le Bihan et al [11], [12] to quantitatively assess the microscopic motions that occur in the sub-voxel scale on MR imaging. It is demonstrated that three parameters, i.e., pure molecular diffusion, microcirculation (or blood perfusion, pseudo-diffusion), as well as their volume fraction, can be distinguished and quantified by fitting the acquired diffusion-weighted (DW) images with multiple *b* values encompassing both low *b* values (<200 sec/mm^2^) and high *b* values (>200 sec/mm^2^) to a bi-exponential decay model. These three IVIM parameters have shown potentials for better tissue characterization than the apparent diffusion coefficient (ADC) derived from the normal mono-exponential diffusion weighted imaging (DWI) model for many clinical applications [13], [14]. Previously, the use of IVIM imaging has been limited to neuroradiologic applications because the abdominal organs can be subject to respiratory and other motion artifacts. The advent of respiratory triggering combined with the use of parallel imaging allows IVIM MR imaging to be applied in the evaluation of the liver in patients with fibrosis. Liver fibrosis is a nonspecific response to chronic liver disease that leads to excess synthesis of extracellular matrix (ECM), especially collagen fibers, in which the protons are less abundant and are tightly bound. Several studies suggested that molecular water diffusion in fibrotic liver would be restricted by the presence of collagen fibers in the distorted lobular structure, and that ADC values decreased in fibrotic liver as compared with normal liver [13], [14]. Given the relatively high blood volume fraction of ≈25–30 mL of blood per 100 g in liver [15], perfusion can contribute to the diffusion measurements significantly because of the incoherent motion of blood in pseudorandom capillary network at the macroscopic level. It is well accepted that liver fibrosis is associated with reduced liver perfusion [15]–[18]. Blood perfusion in chronic liver disease has been recognized as an important marker of liver fibrosis [19]. The purpose of this study was to prospectively evaluate a respiratory triggered DW imaging sequence combined with parallel imaging to determine whether pure molecular-based diffusion coefficient (*D*) and perfusion-related diffusion parameters (perfusion fraction *f*, perfusion-related diffusion coefficient D*) differ in healthy livers and fibrotic livers through intra-voxel incoherent motion (IVIM) MR imaging.

## Material and Methods

This prospective study was approved by The Shenzhen No. 3 People's Hospital Research and Ethics Committee and written informed consent for all participants was obtained. The consent form was also approved by the local Research and Ethics Committee and hard copy was archived in the Department of Radiology of the Shenzhen No. 3 People's Hospital. 17 healthy volunteers (10 males, 7 females, mean age: 36.4-yrs old; range: 21–79 yrs old) and 34 consecutively patients (23 males, 11 females; mean age: 37.3 yrs old; range: 22–57 yrs old) with confirmed liver fibrosis (stage 1 - stage 4) were included. All the patients had hepatitis B virus infection. Liver biopsy and MRI were performed with less than one month interval (range: 0–30 days, median: 12 days), with biopsy performed ahead of MRI. All liver MR imaging examinations were reviewed by two radiologists. Besides liver fibrosis, no patient had liver focal lesions. Biopsy specimen of 10 mm length was taken from each patient and Hematoxylin and Eosin (HE) staining was used. The histopathology results were read in consensus by two pathologists with>10 years' experience. Histopathologically [20], stage 1 of liver fibrosis is mild fibrosis only seen at the portal area; stage 2 indicates fibrosis extending out from the portal areas with rare bridges between portal areas, but without the destruction of the lobular structure; stage 3 of liver fibrosis is severe fibrosis, there is fibrostic bridging between portal areas and between portal areas and center veins; In stage 4 there are pseudo-lobules formed and this stage is the final stage of cirrhosis. Of the 34 patients, 14 had stage-1 liver fibrosis, 8 had stage-2 liver fibrosis, 8 had stage-3 liver fibrosis, and 4 had stage-4 liver fibrosis.

All MR imaging examinations were performed with a 1.5-T MR imaging system (Achieva, Philips Healthcare, Netherlands). The liver MR imaging protocol included transverse T1-weighted gradient-echo sequence, transverse T2-weighted turbo spin-echo sequence, and the transverse IVIM DW imaging sequence. T1-weighted gradient-echo sequence included TR  = 11 ms, TE  = 6.9 ms, Matrix  = 252×151, FOV  = 375 mm×304 mm, slice thickness: 7 mm, slice gap  = 1 mm, Slices 24, NEX  = 1. T2-weighted gradient-echo sequence included TR  = 421 ms, TE  = 80 ms, Matrix  = 268×184, FOV  = 375 mm×297 mm, Slice thickness  = 7 mm, slice gap  = 1 mm, slices  = 24, NEX  = 1.

The IVIM DW imaging sequence was based on a single-shot DW spin-echo type echo-planar imaging sequence, with ten *b* values of 10, 20, 40, 60, 80, 100, 150, 200, 400, 800 sec/mm^2^ respectively. SPIR technique (Spectral Pre-saturation with Inversion-Recovery) was used for fat suppression. Respiratory triggering was performed using an air-filled pressure sensor fixed around the upper part of abdomen for each subject. Respiration waveform was detected and monitored on a gating control screen. The DWI acquisition window was only limited to the expiratory phase while dummied to the inpiratory phase. The respiratory-gating resulted in an average TR of 1500 msec, and the TE was 63 msec. Other parameters included slice thickness  = 7 mm, matrix: 124×97, FOV  = 375 mm×302 mm, NEX  = 2. The IVIM DW imaging was centered around the liver and the number of slices  = 6. The data acquisition time was 6∶42 minutes.

The IVIM signal attenuation is modeled according to the Equation 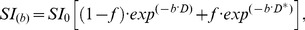
(1) where *SI(b) and SI0* denote the signal intensity acquired with the b-factor value of b and 0, respectively. *f* is the fraction of the pseudo-diffusion linked to microcirculation, *D* is the true diffusion coefficient representing the pure molecular diffusion (slow component of diffusion), and D* is the pseudo-diffusion coefficient representing the incoherent microcirculation within the voxel (perfusion-related diffusion, or fast component of diffusion).

IVIM parameter was quantified using the asymptotic fitting method [21], which was verified to perform better that the normal simultaneous bi-exponential fitting, particularly at relatively low signal-to-noise ratios (SNRs). In detail, considering that D* is significantly greater than *D* due to the much faster capillary blood flow velocity than the water thermal diffusion, its influence on signal decay could be neglected for the *b* factors greater than 200 sec/mm^2^ [4]. Hence, the estimation of *D* was obtained by a least-squares linear fitting of the logarithmized image intensity at the *b* values greater than 200 sec/mm^2^ to a linear equation. The fitted curve was then extrapolated to obtain an intercept at b = 0. The ratio between this intercept and the SI_0_, gave an estimate of the perfusion fraction *f*. Finally, the obtained *D* and *f* were substituted into Eq. [1] and were non-linear least-square fitted against all b-factors to estimate D* using the Levenberg-Marquardt algorithm. When the function failed to converge, pixel values were automatically discarded. To ensure the quantification reliability, the calculated IVIM parameter values for those pixels with the goodness of fit R^2^<0.7 were excluded for the subsequent analysis as well [22]–[23]. R^2^ was defined as 
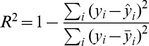
 where 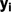 is the measured value, 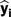 is the corresponding fitted value, and 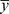 is mean value of measured data values, i.e. 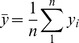.

**Figure 1:**
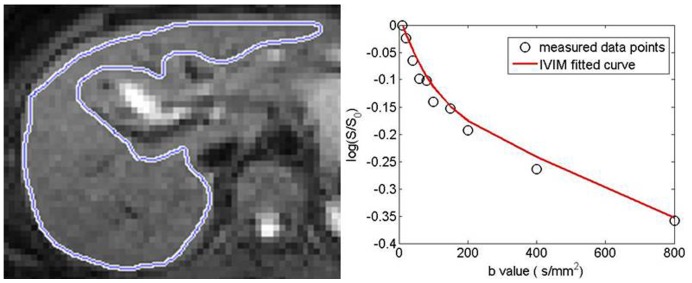
Region of interest (ROI) on the b = 0 diffusion weighted MR image (left) and the corresponding IVIM fitted curve of measured signal (right), showing a bi-exponential decay. The ROI was placed over the whole liver avoiding artifacts and blood.

All curve fitting algorithms were implemented in a home-developed program using MatLab (Mathworks, Natick, MA), which allowed the extraction of parametric maps of *f*, *D*, and D*. All regions-of-interest (ROIs) were manually positioned on the *b* = 0 DW images by a single author who had 5 years' experience reading abdominal MR images. ROIs were positioned to cover a large portion of liver parenchyma while avoiding large vessels (Fig 1), with confirmation provided by visually comparing the ROI positioning on IVIM DW images and on T1-weighted and T2-weighted images. For ROI analysis, the IVIM parameters were calculated based on the mean signal intensity of the whole ROI. Out of the six IVIM DW slices, the central three slices were selected for measurement, and the mean ROI based value of the three slices was regarded as the value of the patient.

Statistical analysis was performed using software SPSS (version 12.0 for Windows; SPSS, Chicago, Ill). The mean value of IVIM parameters as well as standard deviation (SD, related to per subject) of each groups are presented. Mann Whitney U test was used for comparison between the IVIM parameter values obtained from healthy volunteer and patients. One-way analysis of variance (ANOVA) and Bonferroni adjustment for multiple comparisons were used in evaluating the IVIM values from different grades of liver fibrosis. For the comparison between different liver fibrosis stages, stage-3 subjects and stage-4 subjects were grouped together for analysis, due to the limited patient number of stage-4. A *p* value <0.05 was considered significant.

## Results

IVIM DW imaging data were acquired in all subjects. No data suffered from severe motion induced displacements or artifacts hampered IVIM quantification in this study. Curves of IVIM signal-intensity decrease demonstrated biexponential type decay as previously reported [13], regardless of the measurements were obtained in the healthy livers or in the liver fibrotic livers (Fig 1).

**Figure 2:**
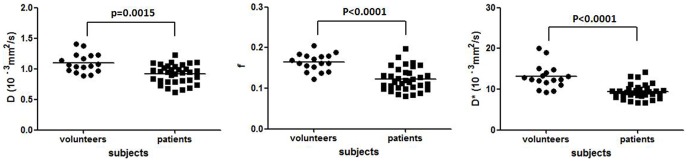
*D, f*, and D* values in healthy livers and fibrotic livers. *D, f*, and D* are significantly lower in fibrotic livers than in healthy livers.

The mean *D, f*, and D* (×10^−3^ mm^2^/s) values measured in healthy volunteers and patients liver were 1.096±0.155 vs 0.917±0.152 (10^−3^ mm^2^/s, p = 0.0015), 0.164±0.021 vs 0.123±0.029 (p<0.0001), and 13.085±2.943 vs 9.423±1.737 (10^−3^ mm^2^/s, p<0.0001) respectively (Fig 2). With *D*, *f*, and D* in fibrotic livers all significantly lower than those in healthy livers. As the fibrosis severity progressed, *D*, *f*, and D* values decreased. A trend towards lower *f* and D* with the increase of fibrosis stage was statistically significant (Table 1, 2).

**Table 1:** Pure molecular diffusion (*D*), perfusion fraction (*f*), and perfusion-related diffusion (D*) values in different stages of fibrotic livers.

Patients	*D* (×10^−3^ mm^2^/s)	*f*	D*(×10^−3^ mm^2^/s)
stage 1 (n = 14)	0.981±0.138	0.145±0.028	10.584±1.872
stage 2 (n = 8)	0.833±0.146	0.119±0.014†	9.028±1.290
stage 3&4 (n = 12)	0.898±0.152	0.100±0.014†	8.332±0.851
*p*-value of ANOVA	0.074	<0.001	0.001
*p*-value of trend	0.154	<0.001	<0.001

† p-value<0.05 comparing stage 2 or stage 3&4 with stage 1, with Bonferroni adjustment for multiple comparisons.

**Table 2:** p-value with Bonferroni adjustment for comparison of pure molecular diffusion (*D*), perfusion fraction (*f*), and perfusion-related diffusion (D*) values in different stages of fibrotic livers.

p-value with Bonferroni adjustment	*D*(×10^−3^ mm^2^/s)	*f*	D*(×10^−3^ mm^2^/s)
stage 1 vs 2	0.083	0.024	0.065
stage 1 vs 3&4	0.462	<0.001	0.001
stage 2 vs 3&4	0.995	0.184	0.903

## Discussion

In patients with early liver fibrosis, the liver parenchyma usually has a normal appearance or may exhibit only subtle, nonspecific heterogeneity on conventional MR images, though later-stage fibrotic liver develops characteristic morphologic alterations such as surface nodularity, widening of fissures, expansion of the gallbladder fossa, notching of the right lobe, atrophy of the right lobe, and relative enlargement of the lateral segments of the left lobe and caudate lobe [24], [25]. A number of MR imaging techniques have been investigated to assess liver fibrosis, including T1rho imaging [22], [23], [26]–[29], double contrast material–enhanced MR imaging [30], gadoxetate disodium-enhanced MRI [31], computer-aided analysis of hepatic contours [32], assessing liver strain using tagged MRI [33], and MR elastography [34], [35]. The IVIM model assumes that each imaging voxel comprises nonexchanging intravascular and extravascular compartments. While the intravascular compartment with perfusion fraction *f* is described by the pseudorandom blood perfusion D*, the extravascular compartment is described by the true molecular diffusion *D*. With low *b*-values, the intravoxel spin dephasing caused by the pseudorandom blood flow in the presence of diffusion gradient will contribute more to the signal attenuation. During the latter part of the signal decay, the attenuation was mainly a consequence of the molecular water diffusion because the blood signal would be mostly suppressed by the large diffusion gradients. Faster signal decay occurred with low b-values in liver due to the contribution from both diffusion and perfusion because of its relatively high blood volume fraction. Recently there are great interests of using IVIM technique to study diffused liver diseases. IVIM MR imaging is a noninvasive technique, it does not require the intravenous injection of contrast agents or the use of additional hardware (such as the external mechanical driver in the case of elastography), also its imaging sequence is readily available on all commercial clinical MRI scanners.

Yamada et al were the first to assess IVIM MRI in abdominal organs, although they used only a limited number of *b*-values (30, 300, 900, and 1100 s/mm^2^), and did not calculate pseudo-diffusion values [36]. They found the ADCs of solid organs and solid lesions were significantly higher than their *D* values, indicating a high contribution of perfusion *f* to the ADCs. With IVIM techniques, Luciani *et al* reported that D* was significantly reduced in the liver fibrosis compared with those in the healthy liver group, but there was no significant difference between *D* and *f* measurements in the healthy liver (n = 25) and the liver fibrosis (n = 12) groups [13]. Guiu *et al* reported that *D* and D* were significantly lower in steatotic compared with nonsteatotic livers. However, *f* was significantly higher in steatotic compared with nonsteatotic livers [37]. In another patient based study, Patel *et al* reported that *f, D* and D* in liver cirrhosis were lower than non-cirrhosis liver, however, no further grading was performed within their liver cirrhosis subjects as only 3 patients (out of 14 patients) had histopathology data [38]. In a rat model of diethylnitrosamine-induced liver fibrosis, Zhang *et al* reported that *f* values decreased significantly with the increasing fibrosis level; but *D* was poorly correlated with fibrosis level [39]. In a carbon tetrachloride induced rat liver fibrosis model, Chow *et al* reported that as liver fibrosis progressed, *D* and D* decreased, however there was no change in *f* [14]. Joo *et al* reported that *f* was significantly lower in rabbits with nonalcoholic fatty liver disease than in those with a normal liver, and it decreased further as severity of nonalcoholic fatty liver disease increased, on the other hand, *D* and D* did not differ significantly between the nonalcoholic fatty liver disease severity groups [40].

In the phantom part of the study Luciani *et al* showed that the signal decay with the IVIM DW imaging sequence was monoexponential, thus suggesting that the biexponential signal decay encountered in vivo cannot be attributed to a systematic error due to the imaging sequence [13], [41]. Our findings confirm that liver diffusion combines both pure molecular diffusion (slow component of diffusion) and capillary perfusion (fast component of diffusion). Liver fibrosis is associated with progressive increase in connective tissue. The increased proportion of collagen fibers is believed to impair Brownian water motion within fibrotic livers. It is well accepted that liver cirrhosis is associated with reduced liver perfusion, particularly with reduced portal flow [13]. Accumulation of collagen deposits and highly contractile activated stellate cells contribute to increased hepatic resistance to portal blood flow, development of portal hypertension, and reduced portal blood perfusion in liver fibrosis. The decreased blood perfusion could arise from a number of concomitant alterations in the tissue microenvironment, including collagen deposition, fatty infiltration, hepatitis, cell necrosis/apoptosis, inflammatory cell infiltration, and fibroblast proliferation with different degrees. Our results demonstrated all *D f*, and D* values were lower in the fibrotic livers compared with the healthy livers, and progressively so as the severity of liver fibrosis increased. To our knowledge, this is the first study such effects are demonstrated in biopsy confirmed human livers in a prospective clinical trial. Compared with the previous patient based studies, our study is characterized by that the coefficient of variation is relatively small, being 0.22 and 0.18 for healthy volunteers and patients respectively, while the patient number was relatively large. Different *f* and D* values have been reported in literature [14,37]–[40]. It is known that IVIM techniques can suffer from measurement reproducibility for *f* and D* values [41]. Further studies with better measurement precision and larger study cohort are necessary to resolve the differences reported in the literature.

There are a few limitations of this study. Only hepatitis B virus related liver fibrosis were included in this study. The study subject number was small for the stage 4 fibrosis group. For this reason, the ability of IVIM DW imaging to help provide a distinction between patients with scores of stage 3 to stage 4 could not be reliably assessed. Statistical power could be further increased with access to larger sample sizes in future studies. This study was carried out using a 1.5 T scanner. Although the image artifacts, e.g., susceptibility induced artifacts, at 1.5 T are considered less than at 3 T, the relatively low signal-to-noise ratio (SNR) may compromise the precision of the calculated IVIM parameters, particularly for *f* and D*. This technical issue was compensated by the NEX of two for acquisition as well as the carefully optimized asymptotic IVIM fitting [21], [42]. It is possible that a higher field 3 T scanner may offer more distinguishing power with the better SNR by taking the advantage of better SNR. In this pilot study, ROIs were manually positioned on the extracted parametric images. The inter-reader measurement reproducibility was not assessed in this study. However, previous study demonstrated excellent inter-reader measurement reproducibility for similar approaches [43]. Future study can employ other sophisticated methods like histogram analysis and texture analysis. IVIM quantification can be affected by the choice of *b*-values used and measurement accuracy at low *b*-value [44]–[46]. Another limitation is that liver fat content has not been quantified in this study. As cirrhosis tends to be coupled with fat accumulation, fatty tissues in liver have low diffusion values [36], [47]. However, another study reported that hepatic steatosis did not affect measurement of perfusion or diffusion and is unlikely to confound the use of apparent diffusivity to evaluate hepatic fibrosis [48]. The extent of perfusion contribution to apparent diffusion measurement can be influenced by the actual imaging sequences and imaging parameters, such as voxel size and the effects of blood flow under a specific sequence setting. Optimizations of acquisition protocol and a post-processing algorithm may minimize the overall measurement errors and hence be more robust for clinical applications. In this study, simultaneous assessment of the traditional contrast enhanced liver perfusion MR imaging was not performed, combining perfusion MR imaging with IVIM imaging would potentially provide additional information. Better improvement of IVIM MR imaging technique will extend its application in other organs and also for therapeutic effect evaluations [49]–[52]

In conclusion, our IVIM MR study shows lower pure molecular diffusion, lower perfusion volume fraction, and lower perfusion-related diffusion in fibrotic livers. Through employing multiple b-values, IVIM analysis has the potential to quantify diffusion and perfusion parameters in liver without the use of contrast agents.
